# Motor Control Deficits in Facial Synkinesis Patients: Neuroimaging Evidences of Cerebral Cortex Involvement

**DOI:** 10.1155/2019/7235808

**Published:** 2019-03-27

**Authors:** Jia-jia Wu, Ye-chen Lu, Mou-xiong Zheng, Xu-yun Hua, Jian-guang Xu, Wei Ding, Chun-lei Shan

**Affiliations:** ^1^Center of Rehabilitation Medicine, Yueyang Hospital of Integrated Traditional Chinese and Western Medicine, Shanghai University of Traditional Chinese Medicine, Shanghai, China; ^2^School of Rehabilitation Science, Shanghai University of Traditional Chinese Medicine, Shanghai, China; ^3^Department of Trauma and Orthopedics, Yueyang Hospital of Integrated Traditional Chinese and Western Medicine, Shanghai University of Traditional Chinese Medicine, Shanghai, China; ^4^Department of Plastic and Reconstructive Surgery, Shanghai Ninth People Hospital Affiliated to Shanghai Jiaotong University School of Medicine, Shanghai, China

## Abstract

**Objective:**

Facial synkinesis is a severe sequelae of facial nerve malfunction. Once the synkinesis is established, it is extremely difficult for patients to recover. Given that the restoration of motor or sensory function after peripheral nerve injury was closely related with cortical plasticity, we investigated cortical plasticity in facial synkinesis patients by the frequency-specific data which remains largely uncharacterized.

**Materials and Methods:**

Resting-state fMRI was conducted in 20 facial synkinesis patients and 19 healthy controls, and the amplitude of low-frequency fluctuation (ALFF) in five different frequency bands (slow-6: 0-0.01 Hz; slow-5: 0.01-0.027 Hz; slow-4: 0.027-0.073 Hz; slow-3: 0.073-0.167 Hz; and slow-2: 0.167-0.25 Hz) was calculated, respectively. And the relationship between ALFF and clinical outcomes was also analyzed.

**Results:**

Comparing with the healthy controls, facial synkinesis patients showed significantly different ALFF values, mainly in the sensorimotor areas. Furthermore, increased ALFF of the ipsilateral insula in the slow-6 band was significantly related with better facial nerve function.

**Conclusion:**

Increased ALFF values in the ipsilateral insula might reflect an abnormal state of hypercompensation in motor control of facial synkinesis patients. It provided valuable spatial information about the functionally aberrant regions, which implied the possible involvement of motor control system in facial synkinesis.

## 1. Introduction

Facial synkinesis, one of the most troublesome sequelae of facial nerve paralysis, is characterized by synchronous and involuntary contractions of the muscles during volitional facial movements [1, 2]. Currently, despite the improved surgical techniques coupled with the use of botulinum toxin A injection, the results of these remained far from satisfactory [3, 4]. In the past years, researchers demonstrated several possible mechanisms of facial synkinesis, including aberrant regeneration and mismatch of axons [1, 5], ephaptic transmission between neighboring axons [5], and overacting of the facial nucleus in the pons [6]. However, none of them has perfectly interpreted all the issues about facial synkinesis. In other words, the underlying pathological mechanism of facial synkinesis is still not clearly elucidated. In the recent years, emerging evidences showed that functional outcome of peripheral neuropathy was closely associated with both peripheral recovery and cortical adaptation [7]. After years of digging peripheral reasons of facial synkinesis, we speculate that brain plasticity is also responsible for the deficits of facial motor control. Therefore, the investigation of cortical plasticity derived from facial synkinesis is now an interesting and vital question.

In our previous studies, we investigated the cortical functional alterations in patients with unilateral facial synkinesis using the task-designed fMRI [8, 9]. Results demonstrated that the distance of the representation sites during ipsilateral blinking and smiling tasks was decreased on the affected half face compared to the unaffected face in patients. And facial movements on either side performed by patients caused more intensive activation in the supplementary motor area on the contralateral side of the affected face than those on the unaffected side. Although we have successfully described alternation of activation in the primary sensorimotor area, higher order processing system, which also contributes to the complex motor control, is still out of our vision. The regulatory effect on facial motor control from other brain regions could not be ignored. There is a possibility that the facial synkinesis is not only a single problem of motor execution but also a complex problem concerning motor regulation or motor control. A resting-state fMRI study indicated that patients with facial palsy showed decreased connectivity mainly in areas responsible for sensorimotor integration and supervision, and the decreased connectivity between SII and the insula significantly correlated to the severity of the facial palsy [10]. Considering the persistency of facial nerve malfunction, some resting-state fMRI measurements such as amplitude of low-frequency fluctuation (ALFF) might provide more valuable information of the brain status.

ALFF is an effective measurement for detecting low-frequency oscillation (LFO) activities at resting state, which has been widely applied in functional neuroimaging [11]. Recent researches have indicated that neuronal oscillations are distributed linearly on the natural logarithmic scale and different spatial distributions of ALFF in distinct frequency bands may result from the neuronal input selection and plasticity [12–15]. However, the combination of every different band of ALFF results in insignificance of statistical analysis. In this study, the low-frequency fluctuation range is subdivided into five frequency bands: slow-6 (0-0.01 Hz), slow-5 (0.01-0.027 Hz), slow-4 (0.027-0.073 Hz), slow-3 (0.073-0.198 Hz), and slow-2 (0.198-0.25 Hz) [15]. And the power of different frequency bands varied among different anatomical and functional properties of the brain. With this method, we aimed to investigate the spontaneous changes of neural activity in facial synkinesis patients by the frequency-specific data, which would lead to a better understanding of facial synkinesis-related neuroplasticity. Furthermore, the relationship between ALFF and clinical outcomes was also analyzed. We also attempted to address the most correlated region and its frequency band, which might offer useful localization advices for researches of cortical interventions.

## 2. Methods and Materials

### 2.1. Participants

A total of 20 facial synkinesis patients and 19 healthy controls were enrolled in this study. Diagnosis of facial synkinesis was made by two experienced plastic surgeons from the Department of Plastic Surgery at The Ninth People's Hospital, School of Medicine, Shanghai Jiaotong University, before the fMRI scans. We recruited patients with unilateral facial synkinesis, absence of nerve transposition, and with no other medical or psychological disorders. Participants were excluded if the facial palsy was recurrent or with concurrent peripheral neuropathy and with contraindications to investigation by MRI. The study was approved by the local ethics committee, and all patients provided written informed consent according to the Helsinki Declaration.

### 2.2. Clinical Assessments

The Sunnybrook facial grading system was applied for the clinical assessment of peripheral facial nerve palsy [16]. This grading system measures three components including facial resting symmetry, symmetry of voluntary movement, and synkinesis. The score ranges from 0 to 100, with higher score indicating better facial nerve function. In this study, the assessment of facial nerve function was directly evaluated by two experienced physicians after the fMRI scan.

### 2.3. fMRI Acquisition

The duration from facial palsy to fMRI scanning is provided in Table 1. The fMRI data were collected using a 3.0T MRI scanner (MR750, GE Healthcare, USA). Participants were instructed to lie still and rest quietly and their heads were immobilized with foam pads. The resting-state fMRI data were obtained with a single-shot gradient-recalled EPI sequence with the following parameters: interleaved scanning order, slice number = 43, matrix size = 64 × 64, FOV = 220 × 220 mm, TR = 2000 ms, flip angle = 90°, slice thickness = 3.2 mm, gap = 0 (voxel size 3.4 × 3.4 × 3.2 mm^3^), and number of acquisitions = 240.

### 2.4. Data Preprocessing

fMRI data were preprocessed in accordance with pipelines recommended by Yu-Feng et al. [17] using the Statistical Parametric Mapping 8 toolbox (http://www.fil.ion.ucl.ac.uk/spm/) on MATLAB 2014a platform. The brain images of right-sided facial synkinesis patients were flipped along the *y*-axis prior to analysis to ensure the consistency of sides among patients. The first 10 images of each functional session were removed for the signal equilibrium. The acquired functional images were preprocessed as follows: (1) slice scan time correction, (2) head movement correction (the head movements were all less than 2.5 mm or 2.5 degrees in any direction), (3) spatial normalization of the functional images via standard EPI template, (4) regressing nuisance variables including the white matter and cerebral spinal fluid blood oxygen level-dependent (BOLD) signal and effects of head motion using six head motion profiles, (5) spatial smoothing using a Gaussian kernel of 4 mm full width at half maximum, and (6) removal of linear trends.

No temporal filtering during preprocessing assures that the entire frequency band below the Nyquist frequency (0.25 Hz) can be examined in subsequent analyses of ALFF.

### 2.5. ALFF Measures in Different Frequency Bands

Subsequent to data preprocessing, the time series for each voxel was transformed to the frequency domain using the fast Fourier transform. The power spectrum was obtained in different frequency bands at each voxel. The low-frequency range was subdivided into five bands as previously defined: slow-6 (0-0.01 Hz), slow-5 (0.01-0.027 Hz), slow-4 (0.027-0.073 Hz), slow-3 (0.073-0.198 Hz), and slow-2 (0.198-0.25 Hz) [15].

As the power of a given frequency was proportional to the square of the amplitude of this frequency component, the square root of the power spectrum was calculated at each frequency. Then, the averaged square root (i.e., ALFF value) of the power spectrum in different frequency bands was calculated [18]. Finally, each individual's ALFF value was transformed to *Z* score to allow further comparison between groups.

### 2.6. Statistical Analysis

To investigate the effects of disease on different frequency bands, we performed an ANOVA (flexible factorial design, 2 × 5) using the SPM8 software within a standard (Montreal Neurological Institute space) grey matter mask. The group was considered to be a between-subject factor with two levels (facial synkinesis patients vs. healthy controls). The frequency band was regarded as a correlated repeated measure factor with five levels. The interactions of group and frequency were considered as effects in the model. After the interaction effect analysis, significant clusters were generated within a binary mask. Further, post hoc two-sample *t*-tests were performed for group comparison of each frequency band within the previously generated mask. All significance tests were conducted at a false discovery rate (FDR) threshold of *P* = 0.05.

### 2.7. Correlation Analysis

The correlation between ALFF values of the ROIs in each frequency band and Sunnybrook scores was also analyzed. The ROIs for correlation analysis were extracted from the largest significant cluster revealed by post hoc *t*-tests between the two groups in each frequency band. Spearman's correlative analyses were used to examine relationships between abnormal ALFF values and Sunnybrook scores in facial synkinesis patients using SPSS 22 software (SPSS Inc., Chicago, IL).

## 3. Results

### 3.1. Clinical Characteristics

There were no significant differences in age or gender between the facial synkinesis patients and the healthy controls. Among the facial synkinesis patients, 9 were with right-sided palsy and 11 were left-sided. The duration of facial palsy from onset to fMRI scanning was 26.05 ± 14.20 months. All patients showed a unilateral loss of facial function, with mean Sunnybrook score of 42.45 ± 19.24 points (Table 1).

### 3.2. ALFF Results

#### 3.2.1. Interaction Effects of Group × Frequency Band

**Figure 1:**
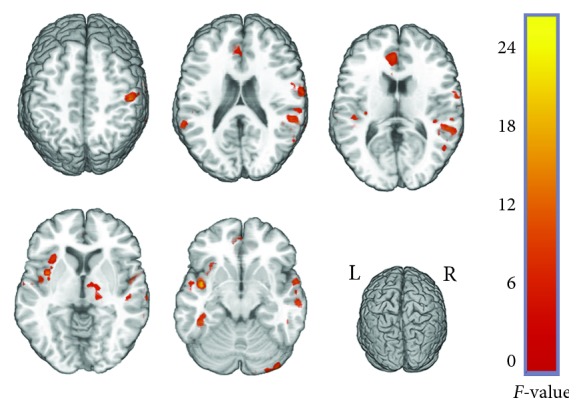
Interaction effects of group × frequency by a two-way ANOVA. The red-yellow regions represent significant interaction between disease and frequency.

Significant interaction effects of disease × frequency were distributed over the left insula, right middle temporal cortex, left inferior temporal cortex, right cerebellum, bilateral superior temporal cortex, bilateral orbit frontal cortex, right postcentral cortex, right thalamus, and left anterior cingulate cortex (ACC) (Figure 1 and Table 2).

#### 3.2.2. Post hoc Two-Sample *t*-Tests between Groups

**Figure 2:**
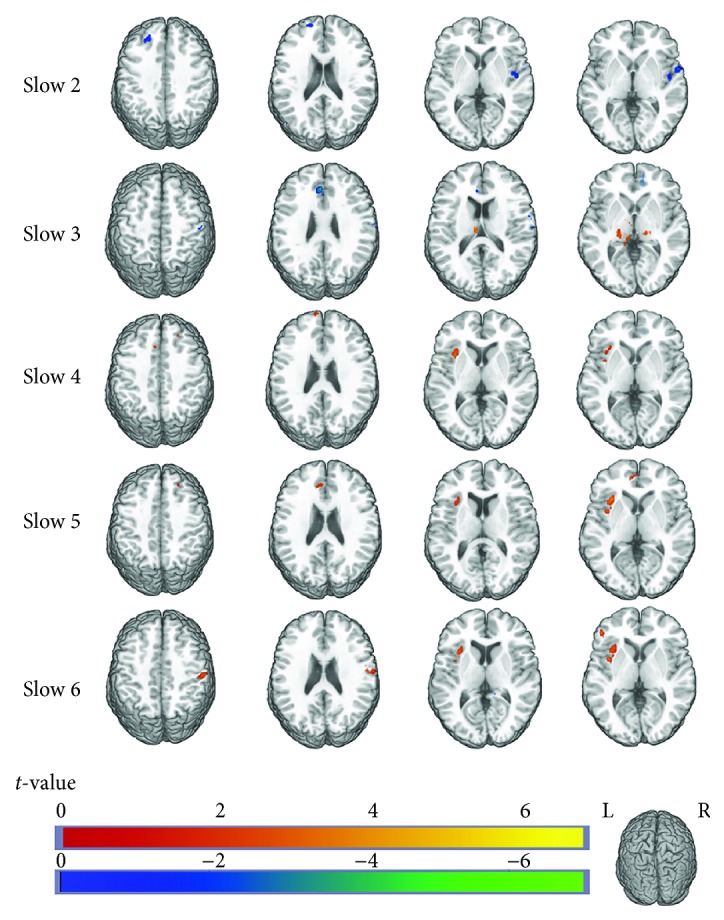
Difference in the amplitude of low-frequency fluctuation (ALFF) between facial synkinesis patients and healthy controls. Each row in the figure displays difference of ALFF between facial synkinesis patients and healthy controls in each of the five frequency bands. The warm tone represents signal of patients greater than healthy controls, while the cold tone represents signal of patients less than healthy controls.

To investigate differences between the two groups in each frequency band, further post hoc two-sample *t*-tests were performed (Figure 2 and Table 3).

In the slow-2 band, facial synkinesis patients exhibited significantly decreased ALFF values comparing with the healthy controls in the bilateral ACC, right insula, right cerebellum, right superior temporal cortex, left superior frontal cortex, right middle temporal cortex, right postcentral cortex, and right medial superior frontal gyrus.

In the slow-3 band, facial synkinesis patients showed significantly increased ALFF in the left hippocampus, left cerebellum, and bilateral thalamus and significantly decreased ALFF in the left ACC, right middle temporal cortex, right cerebellum, right postcentral cortex, and right orbit frontal cortex.

In the slow-4 band, facial synkinesis patients showed significantly increased ALFF in the right cerebellum, left insula, right superior temporal cortex, left hippocampus, left medial superior frontal cortex, and right superior frontal cortex and significantly decreased ALFF in the right orbit frontal cortex.

In the slow-5 band, facial synkinesis patients showed significantly increased ALFF in the left insula and left inferior temporal cortex, right superior frontal cortex, left ACC, and left hippocampus and significantly decreased ALFF in the right hippocampus.

In the slow-6 band, facial synkinesis patients showed significantly increased ALFF in the left insula, left inferior frontal cortex, left inferior temporal cortex, left middle temporal cortex, right postcentral cortex, left ACC, and right middle temporal cortex and significantly decreased ALFF in the right hippocampus, and left cerebellum.

### 3.3. Clinical Correlation

**Figure 3:**
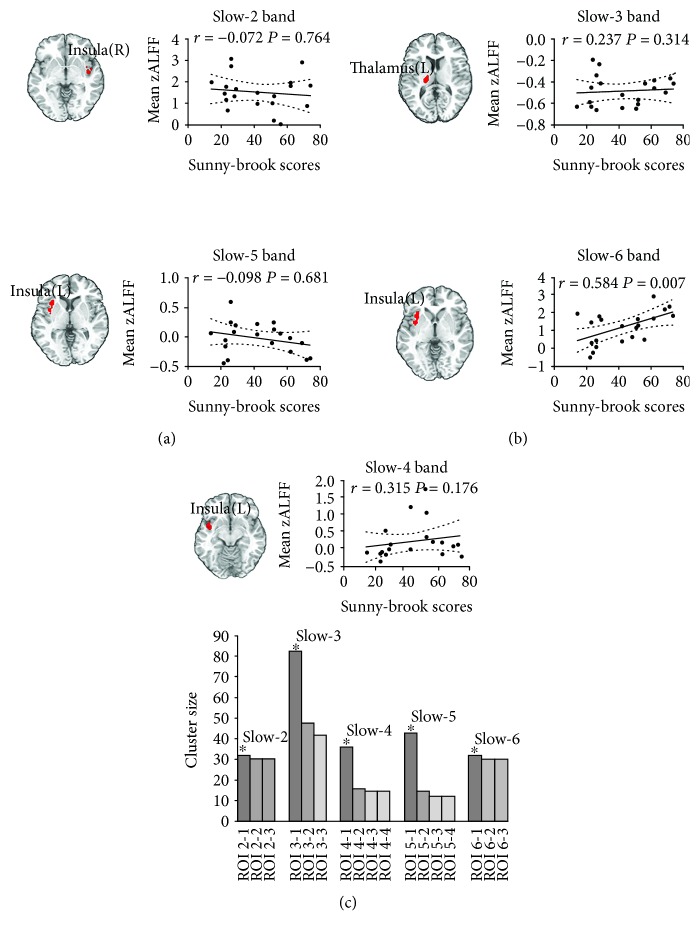
Spearman correlation analysis between Sunnybrook scores and ALFF values in five ROIs. The ROIs were extracted from the largest significant cluster revealed by post hoc *t*-tests between the two groups in each frequency band. The bar graph showed the significant clusters of different ALFF between facial synkinesis patients and healthy controls by a post hoc two-sample *t*-test in each frequency band. ROI 2-1: right insula; ROI 2-2: right superior temporal pole; ROI 2-3: right anterior cingulate cortex(ACC); ROI3-1: left thalamus; ROI3-2: left hippocampus; ROI3-3: right medial orbit frontal cortex; ROI4-1: left insula; ROI4-2: left hippocampus; ROI4-3: right cerebellum; ROI4-4: right superior temporal pole; ROI5-1: left insula; ROI5-2: right hippocampus; ROI5-3: right superior frontal gyrus; ROI5-4: left ACC; ROI6-1: left insula; ROI6-2: right postcentral gyrus; ROI6-3: left middle temporal pole.

Significant correlation was found between the Sunnybrook scores and ALFF values in facial synkinesis patients for the cluster of the left insula in the slow-6 band (*r* = 0.584, *P* = 0.007). However, ALFF values of the other significantly different clusters between groups in the slow-2 to slow-5 bands (the right insula in the slow-2 band, the left thalamus in the slow-3 band, the left insula in the slow-4 band, and the left insula in the slow-5 band) failed to show significant correlation with the Sunnybrook scores (Figure 3).

## 4. Discussion

### 4.1. Main Findings

In the present study, we investigated frequency-specific ALFF alteration between facial synkinesis patients and healthy controls in the distribution of intrinsic brain hemodynamic oscillations across five frequency bands (slow-2 to slow-6). We also investigated the capability of the frequency-specific alterations in providing predictive information with the frequency-specific alterations. The main findings were concluded as follows: (1) a significant interaction effect of the group × frequency band was observed mainly in the sensorimotor regions; (2) post hoc two-sample *t*-tests showed significant differences in ALFF between the two groups in each frequency band; and (3) in the slow-6 band, ALFF values of facial synkinesis patients' insula were most significantly related with facial synkinesis patients' Sunnybrook scores.

### 4.2. Alterations of ALFF in Each Frequency Band

ALFF was reported to describe the local spontaneous neuronal activity [19]. Previous studies of electroencephalography have demonstrated that LFO represented spontaneous neural activity which generated its rhythmic activity patterns through information exchange with neighboring brain areas [15]. Most fMRI studies measured ALFF at a typical frequency range between 0.01 and 0.08 Hz which was thought to be physiologically meaningful [20, 21]. However, the fMRI scan objectively enabled possible detection of fluctuations over 0.1 Hz [21]. According to the Buzsáki framework, the full frequency range (0-0.25 Hz) was subdivided into five frequency bands (slow-2, slow-3, slow-4, slow-5, and slow-6 bands) [15]. Although the exact mechanism of different bands is still controversial, several researchers have tried to give some explanations of frequency-specific ALFF. The neurophysiological mechanisms that underlie oscillations within specific classes may be linked to specific neurophysiological processes, including input selection, plasticity, binding, and consolidation [13–15].

Although the slow-2 and slow-3 bands were typically discarded as physiological noises [14], they were still adopted in the correlation analysis for objective and comprehensive comparison in this study. In these two bands, a significant difference was found in several areas. However, further analysis failed to show significant correlation between Sunnybrook scores and ALFF values in facial synkinesis patients in the most significantly different cluster between the two groups (the right insula in the slow-2 band and the left thalamus in the slow-3 band).

Of note, the slow-4 and slow-5 bands were thought to be linked with neurophysiological processes, which were typically utilized in resting-state fMRI studies (below 0.1 Hz) [20, 21]. In the results of the slow-4 and slow-5 bands, we observed decreased ALFF values for neuronal activity in facial synkinesis patients in the right superior frontal cortex and right hippocampus and increased ALFF values predominantly in areas acting sensorimotor integration functions. Similar with the pattern of ALFF alternations in these two bands, the data analysis in the slow-6 band showed that facial synkinesis patients exhibited significantly increased ALFF values in areas of sensorimotor integration and bilateral temporal lobe, whereas it was decreased in the left right hippocampus and left cerebellum. Based on the results of post hoc two-sample *t*-test, ALFF values of the left insula showed the most significant difference between the two groups in these three frequency bands. And the ALFF value in the slow-6 band was most significantly correlated with Sunnybrook scores. Previous studies demonstrated that the slow-6 band was ascribed to low-frequency drifts [14, 19]. Although a few studies have addressed the indicative utilization of fluctuations in the slow-6 band, our data demonstrated its potential role of neurophysiological biomarker in the functional assessment of facial synkinesis patients.

### 4.3. Insula and Motor Control

This study demonstrated that facial synkinesis was mostly affected in areas of higher order processing sensorimotor information, including the postcentral cortex, insula, thalamus, ACC, hippocampus, and cerebellum. However, the patterns of ALFF alteration in the ACC or cerebellum areas were not fixed. It suggested that these changes were very likely to be caused by the motor-sensory mismatch due to the substantial prediction error. Among those heterogeneous results, it was interesting to find significant difference of ALFF in the insula between the two groups in each frequency band. Moreover, the ALFF values of the left insula in facial synkinesis patients in the slow-6 band were found to correlate with the improvement of facial nerve function significantly. The insula was demonstrated to contribute to hand and eye motor movement in motor control [22, 23]. The result indicated the motor control deficits in facial synkinesis. Recent neuroimaging studies have showed that the insula was associated with higher cognitive processing [24], pain, and sensorimotor processing [25, 26]. The insula participates in higher order processing of somatosensory input and has the most connections with other regions in the brain network, including the thalamus, hippocampus, and ACC [27–29]. In addition to pain, electrical stimulation of the human insula cortex elicits auditory, vestibular, vegetative, olfactory, and gustatory responses, thereby implying a multimodal role for the insula [30]. In the present study, the increased ALFF value in the insula ipsilateral to the lesioned side is considered to be in an abnormal state of hypercompensation in motor control of facial synkinesis patients.

### 4.4. Possible Source of Artifacts

Many studies paid attention to physiological signals arising from cardiac- and respiratory-related processes, for example, heart rate, respiration sources, and blood pressure, which could impact regional measures of ALFF [31–34]. These physiological factors that are closely related with the quality of BOLD signals raise great concerns regarding test-retest reliability [35]. However, Zuo et al. have confirmed test-retest reliability of ALFF [14]. Baria et al. also demonstrated that it showed little difference from uncorrected ones when regressing out cardiac and respiration signal in each subject per voxel [36]. Besides, the noise was generally assumed a random factor in both groups. Thus, there should not be significant differences between the two groups, even when comparing the noise effect among the five frequency bands.

### 4.5. Limitations

This study was an explorative neuroimaging study, and several limitations should be noted. Firstly, the sample size was relatively small, which might have limited the power of persuasion. Secondly, it might be better to compare patients who have recovered from facial paralysis and those who suffered from facial synkinesis to investigate alterations of spontaneous activity caused by facial synkinesis only, although the functional activities in the related brain regions gradually recovered to the normal level with the gradual recovery of facial nerve function [37–39]. Considering unavailability of people recovered from facial paralysis, we recruited healthy normal control instead. Thirdly, the gender and age in the healthy controls did not match the patients perfectly. Finally, the variety of the disease duration was not well controlled, which could bring negative impact on homogeneity within the patient group.

## 5. Conclusion

We reported multiple changes of band-specific ALFF in facial synkinesis patients. The increased ALFF value in the slow-6 band in the ipsilateral insula was positively related with facial nerve function (Sunnybrook scores). This study revealed frequency-specific changes in local spontaneous neuronal activity for severity correlation in facial synkinesis, which could be a potential indicator of disease progression. It also provided valuable spatial information about the functionally aberrant regions, which implied the possible involvement of motor control system in facial synkinesis. Although it is with limitation, this study may broaden our vision of the neural mechanism of facial synkinesis.

## Figures and Tables

**Table 1:** Demographic information of enrolled subjects.

Items	Facial synkinesis group (*n* = 20)	Healthy control group (*n* = 19)	*P* value
Age	33.35 ± 6.97	33.21 ± 6.75	0.950^a^
Gender	2 (female)/18 (male)	2 (female)/17 (male)	0.957^b^
Duration	26.05 ± 14.20 m	—	—
Affected side	9 (right)/11 (left)	—	—
Sunnybrook scores	42.45 ± 19.24	—	—

^a^Two independent sample *t*-tests. ^b^Chi-square test.

**Table 2:** Interaction effects of group × frequency by a two-way ANOVA.

Brain regions	Cluster size	Cluster centroid (MNI)			*F* value
		*x*	*y*	*z*	
Frontal_Med_Orbital_L	39	0	36	-12	24.4579
Insula_L	36	-42	3	-9	25.9851
Postcentral_R	36	51	-27	51	20.6439
Temporal_Sup_R	35	54	-39	15	15.8532
Cingulate_Ant_L	32	-3	36	24	12.3385
Insula_L	31	-30	21	6	9.2927
Temporal_Inf_L	24	-42	-39	-15	16.4087
Cerebellum_Crush1_R	18	39	-87	-33	21.6993
Temporal_Mid_R	18	60	-3	-18	15.6285
Postcentral_R	17	66	-12	21	13.1904
Orbital_frontal_med_R	16	15	48	-18	11.895
Thalamus_R	14	21	-21	6	12.5973
Orbital_frontal_post_L	13	-21	24	-18	11.873
Temporal_Pole_Mid_R	13	45	12	-30	10.9921
Temporal_Mid_R	12	69	-48	6	15.2749
Temporal_Sup_L	12	-57	-45	21	15.1745
Temporal_Pole_Sup_R	11	30	9	-24	20.7315
Temporal_Inf_L	11	-33	12	-39	18.3323

**Table 3:** Differences in ALFF between facial synkinesis patients and healthy controls in 5 frequency bands.

Brain regions		Cluster size	Cluster centroid(MNI)			*t* value
			*x*	*y*	*z*	
Slow-2 band						
Facial synkinesis < healthy controls						
Insula_R		32	48	-6	3	-3.4274
Temporal_Pole_Sup_R		30	30	18	-27	-2.6361
Cingulate_Ant_R		30	9	42	0	-3.3372
Postcentral_R		27	51	-27	48	-3.3814
Cerebelum_Crus1_R		25	39	-87	-33	-4.0931
Temporal_Mid_R		22	60	-3	-18	-2.4222
Frontal_Sup_Medial_R		21	6	36	42	-2.899
Temporal_Pole_Mid_R		19	48	9	-30	-2.8583
Frontal_Sup_L		16	-21	36	36	-2.6557
Cingulate_Ant_L		12	-6	30	18	-3.1261

Slow-3 band					
Facial synkinesis > healthy controls					
Thalamus_L	82	-18	-27	-3	2.8462
Hippocampus_L	48	-21	-24	-9	4.9978
Cerebelum_10_L	30	-21	-33	-45	4.8498
Thalamus_R	11	12	-24	3	2.8505
Facial synkinesis < healthy controls					
Frontal_Med_Orbital_R	42	12	48	-9	-2.7222
Cingulate_Ant_L	22	-3	36	27	-3.8034
Cerebelum_Crus1_R	22	39	-87	-33	-3.2934
Temporal_Mid_R	10	60	-3	-21	-3.3251
Postcentral_R	10	66	-15	15	-3.1111
Cerebelum_Crus2_R	10	39	-75	-51	-2.7581

Slow-4 band					
Facial synkinesis > healthy controls					
Insula_L	36	-27	12	-21	2.5823
Hippocampus_L	16	-21	-24	-9	3.222
Cerebelum_9_R	15	12	-48	-54	4.2794
Temporal_Pole_Sup_R	15	30	9	-24	4.137
Frontal_Sup_Medial_L	12	-9	66	9	3.6189
Hippocampus_L	10	-18	-12	-12	3.9069
Frontal_Sup_2_R	10	24	36	42	2.7278
Facial synkinesis < healthy controls					
Frontal_Med_Orbital_R	10	12	42	-6	-2.3628

Slow-5 band					
Facial synkinesis > healthy controls					
Insula_L	43	-33	15	9	3.353
Frontal_Sup_2_R	12	27	9	66	2.9628
Cingulate_Ant_L	12	-3	33	-9	2.8405
Temporal_Inf_L	11	-42	6	-42	3.2313
Hippocampus_L	11	-21	-24	-9	2.6231
Frontal_Sup_2_R	10	24	39	42	2.3045
Facial synkinesis < healthy controls					
Hippocampus_R	15	33	-12	-15	-2.9439

Slow-6 band					
Facial synkinesis > healthy controls					
Insula_L	61	-39	6	0	3.8307
Postcentral_R	37	57	-21	45	3.3997
Temporal_Pole_Mid_L	28	-21	9	-36	2.6843
Cingulate_Ant_L	27	-3	39	18	3.3037
Temporal_Inf_L	22	-42	6	-42	3.6417
Frontal_Inf_Triangle_L	10	-48	39	3	3.9693
Facial synkinesis < healthy controls					
Cerebelum_9_L	24	-9	-54	-51	-2.8445
Hippocampus_R	15	30	-21	-15	-3.0374

## Data Availability

The data that support the findings of this study are available from the corresponding author, Jian-guang Xu, upon reasonable request.
